# Metabolic Responses of Melanocytes and Melanoma Cells to UVA Radiation and Phytocannabinoids Exposure

**DOI:** 10.3390/antiox15060690

**Published:** 2026-05-30

**Authors:** Michał Biernacki, Ernest Gieniusz, Agnieszka Gęgotek, Morana Jaganjac, Elżbieta Skrzydlewska

**Affiliations:** 1 Department of Analytical Chemistry, Medical University of Bialystok, Mickiewicza 2D, 15-222 Białystok, Poland; 2 Laboratory for Oxidative Stress, Division of Molecular Medicine, Ruder Boskovic Institute, Bijenicka 54, 10000 Zagreb, Croatia; morana.jaganjac@irb.hr

**Keywords:** melanocytes, melanoma cells, UVA radiation, cannabigerol, cannabidiol, oxidative stress, inflammation

## Abstract

Ultraviolet A (UVA) radiation disrupts the redox balance of melanocytes and may lead to the development of melanoma, highlighting the need for new skin protection strategies. This study assessed the effect of phytocannabinoids [cannabigerol (CBG), cannabidiol (CBD), and CBG + CBD] on redox homeostasis in control and UVA-exposed melanocytes and in melanoma cells (SK-Mel-5). UVA radiation increased the activity of prooxidant enzymes in both melanocytes and SK-Mel-5 cells and, consequently, the level of reactive oxygen species (ROS) (approx. 2-fold). It also activated nuclear factor erythroid 2 (Nrf2), as reflected by increased expression of heme oxygenase 1 (HO-1) (melanocytes approx. 2-fold; SK-Mel-5 approx. 7-fold). Concomitantly, antioxidant mechanisms were impaired, as demonstrated by reduced superoxide dismutase (SOD1/SOD2) activity and impaired glutathione and thioredoxin function. These changes were accompanied by increased levels of oxidative damage markers (isoprostanes, 4-hydroxynonenal-4-HNE, and 4-HNE-protein adducts) (43–100%) and increased inflammatory signaling, including increased expression of nuclear factor kappa B (NF-κB) subunits (melanocytes: p52 ~2-fold, p65 ~75%; SK-Mel-5: ~4–4.5-fold) and tumor necrosis factor alpha (TNF-α; ~30%). Phytocannabinoid treatment modulated these UVA-induced changes. In SK-Mel-5 cells, phytocannabinoids normalized the activity of prooxidant enzymes and consequently reduced ROS levels (~30%). They also reduced Nrf2 activation and HO-1 expression; however, CBG increased HO-1 level in melanocytes (~25–40%). Furthermore, phytocannabinoids enhanced antioxidant defense by increasing SOD activity, particularly in melanocytes (~10–40%), and restoring the glutathione and thioredoxin systems. Markers of oxidative damage were reduced by approximately 23–37% after treatment. Furthermore, phytocannabinoids attenuated NF-κB activation (p52 ~18–28%, p65 ~25–29% in melanocytes; ~20% in SK-Mel-5), while TNF-α levels remained unchanged. The effects in non-irradiated cells were modest (<15%). These results suggest that phytocannabinoid-mediated modulation of redox balance may stabilize melanocytes exposed to UVA radiation and potentially reduce the risk of neoplastic transformation. However, the observed protective effects in SK-Mel-5 cells require further investigation and detailed molecular analysis.

## 1. Introduction

Melanocytes are specialized pigment-producing cells localized predominantly within the basal layer of the epidermis. Their primary function is the synthesis of melanin, a photoprotective pigment that mitigates the deleterious effects of ultraviolet (UV) radiation on the skin [1]. Under physiological conditions, melanocyte proliferation and melanin synthesis are tightly regulated by a complex network of signaling pathways involving keratinocytes, fibroblasts, and immune cells [2]. However, both genetic predisposition and environmental stressors, most notably excessive UV exposure, can perturb this regulatory equilibrium, leading to DNA damage, accumulation of oncogenic mutations, and aberrant melanocytic proliferation [3].

Melanoma, which arises from the malignantly transformed melanocytes, represents the most aggressive form of cutaneous malignancy, distinguished by its high metastatic propensity, extensive genomic heterogeneity, and resistance to conventional therapies. Despite continuous advancements in photoprotection and public health initiatives, the global incidence of melanoma continues to rise. Although melanomas constitute approximately 1% of all skin cancers, they account for the majority of skin cancer-related mortality [4]. Unlike most other cancers, melanoma frequently affects young individuals [5]. Advanced melanoma remains highly aggressive, with a strong tendency to metastasize, and only 15–30% of patients with advanced disease achieve a five-year survival [6]. Moreover, melanoma exhibits one of the highest mutation frequencies among human malignancies [7].

The pathogenesis of UV-induced cutaneous malignancies results from a multistep cascade of molecular perturbations. UV radiation promotes excessive generation of reactive oxygen species (ROS), leading to oxidative stress and structural and functional impairment of cellular macromolecules, including lipids, proteins, and nucleic acids [3,8]. These alterations drive the activation of inflammatory pathways, apoptosis, the p53-dependent DNA damage response, increased mutational load, and immunosuppression [3]. Although contemporary therapeutic modalities such as targeted therapy and immune checkpoint inhibitors have markedly improved clinical outcomes, their efficacy remains constrained by adverse effects, tumor heterogeneity, metastatic progression, and rapidly emerging resistance mechanisms [9]. As a result, current research efforts are increasingly directed toward identifying novel molecular targets, preventing melanoma initiation and progression, and exploring adjunctive therapeutic approaches involving bioactive natural compounds with low toxicity [10].

Among such compounds, phytocannabinoids have emerged as promising candidates owing to their ability to modulate cellular processes through well-established regulatory mechanisms [11]. Phytocannabinoids are naturally occurring compounds produced by plants, most notably by *Cannabis sativa* and *Cannabis indica*. They are synthesized in the glandular trichomes of these plants, particularly in the flowers, but can also be found in smaller amounts in leaves and stems [11,12]. Their anticancer activity has been documented across multiple malignancies, including breast, prostate, lung, skin, pancreatic, and bone cancers, as well as lymphoma and glioma [11]. Experimental evidence from diverse in vitro and in vivo models suggests that phytocannabinoids exert antineoplastic effects by modulating cell proliferation, apoptosis, and tumor progression, in part through the regulation of angiogenesis, immune responses, and oncogenic signaling networks [12].

**Figure 1:**
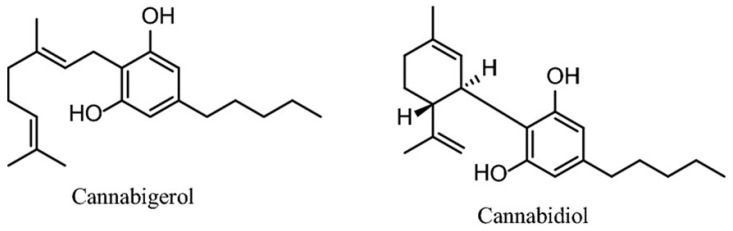
Structures of cannabigerol and cannabidiol.

Cannabidiol (CBD), one of the most extensively investigated nonpsychoactive phytocannabinoids, exerts antiproliferative and proapoptotic effects through modulation of multiple signaling pathways, including transient receptor potential (TRP) channels, peroxisome proliferator-activated receptors (PPARs), and components of the endocannabinoid system [13]. Additionally, CBD suppresses cancer cell invasiveness by altering nuclear factor kappa-light-chain-enhancer of activated B cells (NF-κB), extracellular signal-regulated kinase/protein kinase B (ERK and AKT) signaling, and pathways associated with matrix metalloproteinases and angiogenesis [12]. Although several studies have examined CBD-induced alterations in melanoma cell proliferation, apoptosis, and tumorigenicity [14,15], its potential impact on melanoma cell redox homeostasis remains insufficiently characterized. Another phytocannabinoid of emerging scientific interest is cannabigerol (CBG), whose anticancer effects have been only partially explored in human and murine melanoma models [16]. Notably, CBG has been reported to reduce macrophage colony-stimulating factor 1 (CSF-1) secretion in melanoma cells, reprogram myeloid cell populations, restore CD8^+^ T-cell activity, and potentiate the efficacy of anti-PD-L1 immunotherapy, thereby underscoring its relevance as an immunomodulatory antineoplastic agent [16]. A comparison of the chemical structures of cannabidiol and cannabigerol is presented in Figure 1.

We hypothesize that CBD and CBG differentially modulate UVA-induced redox responses in melanocytes and melanoma cells (SK-Mel-5), involving nuclear factor erythroid 2-related factor 2 (Nrf2) and NF-κB signaling pathways. Therefore, the present study was designed to compare the protective and modulatory effects of CBD and CBG administered individually and in combination on the redox homeostasis of normal melanocytes, and to evaluate their potential therapeutic utility in SK-Mel-5 cells, with particular emphasis on metabolic alterations elicited by UVA irradiation.

## 2. Materials and Methods

### 2.1. Materials and Reagents

All cell lines used in this study were obtained from the American Type Culture Collection (ATCC, Manassas, VA, USA). Human primary epidermal melanocytes (ATCC PCS-200-012) were isolated from neonatal foreskin, while human melanoma cells (SK-Mel-5; ATCC HTB-70) were derived from the skin tissue of a 24-year-old Caucasian female diagnosed with malignant melanoma. The authenticity and purity of all cell lines, validated by STR profiling, were verified by the supplier. Cells were cultured according to ATCC recommendations.

All chemicals and reagents were of analytical grade and obtained from commercial sources. Krebs/HEPES buffer (pH 7.4), 1-hydroxy-3-methoxycarbonyl-2,2,5,5-tetramethylpyrrolidine (CMH), deferoxamine methanesulfonate salt (DF) and sodium diethyldithiocarbamate trihydrate (DETC) were purchased from Noxygen GmbH (Elzach, Germany); cannabidiol (CBD), cannabigerol (CBG) penicillin-streptomycin, phosphate-buffered saline (PBS), NADPH, lucigenin, EGTA, EDTA, sucrose, xanthine, adrenaline, carbonate buffer, sodium dodecyl sulfate (SDS), 5,5′-dithiobis(2-nitrobenzoic acid) (DTNB), reduced glutathione (GSH), oxidized glutathione, KCl, 3,3′,5,5′-tetramethylbenzidine (TMB), hydrogen peroxide (H_2_O_2_), sulfuric acid (H_2_SO_4_), 5-sulfosalicylic acid (SSA), NaOH, sodium borate, Tween 20, pentafluorobenzyl hydroxylamine (PFB-hydroxylamine), benzaldehyde-d6, methanol, ethanol, hexane, and N,O-bis(trimethylsilyl)trifluoroacetamide with 1% trimethylchlorosilane (BSTFA + TMCS) were obtained from and fetal bovine serum was obtained from Thermo Fisher Scientific (Waltham, MA, USA). The thioredoxin reductase assay kit was purchased from Sigma-Aldrich (St. Louis, MO, USA). Primary anti-thioredoxin antibody was obtained from Abcam (Cambridge, MA, USA), while goat anti-rabbit secondary antibody was purchased from Dako. Antibodies against NF-κB (p65 and p52) and TNF-α were obtained from Sigma-Aldrich (St. Louis, MO, USA). Antibodies against heme oxygenase-1 (HO-1) and phospho-Nrf2 (Ser40), as well as anti-4-hydroxynonenal (4-HNE)-protein adduct antibody, were purchased from Invitrogen (Waltham, MA, USA). The EnVision+ Dual Link System-HRP detection kit was obtained from Agilent Technologies (Santa Clara, CA, USA). The following proteins were analyzed: NF-κB(p65) [standard from OriGene (Rockville, MD, USA)], TNF-α [standard from Merck (Darmstadt, Germany)], NF-κB (p52) [standard from LSBio (Seattle, WA, USA)], HO-1 [standard from Enzo Life Sciences (Farmingdale, NY, USA)], and phospho-Nrf2 (Ser40) [standard from Gentaur (Kampenhout, Belgium)]. The internal standard of prostaglandin PGF2α [8-iso-PGF2α-d4] was purchased from Cayman Chemical (Ann Arbor, MI, USA).

### 2.2. Cell Culture and Treatment

Both cell lines were handled according to the supplier protocol. Melanocytes were cultured in Dermal Cell Basal Medium (DCBM; ATCC PCS-200-030) supplemented with Melanocyte Growth Kit (ATCC PCS-200-041) containing 5 µg/mL insulin, 50 µg/mL ascorbic acid, 6 mM L-glutamine, 1 µM epinephrine and 0.2 mM calcium chloride [17] and SK-Mel-5 cells were maintained in Eagle’s Minimum Essential Medium (EMEM; ATCC 30-2003) supplemented with 10% fetal bovine serum [18]. Additionally, both media were protected against bacterial contamination by 50 U/mL penicillin and 50 μg/mL streptomycin. Cells were cultured in 100 mm adherent sterile plastic plates (Sarstedt, Numbrecht, Germany). It is estimated that under experiment, there were approximately 2–4 mln cells on the plate (depending on the line) incubated in 10 mL of medium at 37 °C in a humidified atmosphere containing 5% CO_2_.

When melanocytes (passage 3) and SK-Mel-5 cells (passage 12) reached approximately 90% confluence, they were subjected to further treatment. The cells were washed with phosphate-buffered saline (PBS) and then exposed to UVA radiation (365 nm) at a dose of 18 J/cm^2^, corresponding to 75 ± 5% cell viability based on MTT assay results [19] as described below. Melanocytes and SK-Mel-5 cells were irradiated in plastic dishes surrounded by ice, positioned 15 cm below five (6 W) UVA lamps (365 nm; Bio-Link Crosslinker BLX 365, Vilber Lourmat, (Marne-la-Vallée, France)), delivering an intensity of 4.08 mW/cm^2^. The buffer temperature during exposure did not exceed 10 °C to avoid the heating cells during irradiation, as well as inhibit the activity of proteolytic and metabolic enzymes during incubation in PBS buffer. The total irradiation time was 70 min, during which non-irradiated control cells were incubated under identical conditions in the dark to protect them from UVA radiation.

CBD and CBG (Sigma-Aldrich, St. Louis, MO, USA) stock solutions were prepared in ethanol to achieve a 0.3% ethanol concentration when added to the cell medium. Therefore, ethanol was also added to the medium of control cells at the same concentration. CBD (5 µM), CBG (1 µM), and their combination (5 µM CBD + 1 µM CBG) were added to the medium of control and UVA-irradiated cells, and the cells were incubated for 24 h. The concentrations of these compounds were selected as the highest ones that do not lower viability of non-irradiated cell measured using MTT assay [20]. The details of MTT assay performed for selected CBD and CBG concentrations is described below. Moreover, the chosen concentrations of CBD and CBG do not alter the morphology of the tested non-irradiated cells as visualized using NikonEclipse Ti microscope combined with MagingSource camera operated by Nikon’s NIS-Elements imaging software version 5.30.02 (Nikon Instruments Inc., Melville, NY, USA) (Figure S1).

Following experimental treatment, cells were washed twice with PBS to remove residual medium and harvested by scraping. The collected cells were centrifuged (300× *g*, 5 min, 4 °C), and the resulting pellets were re-suspended in PBS. Cells were sonicated on ice (short pulses) to obtain whole-cell lysates. The lysates were then centrifuged at 10,000× *g* for 10 min at 4 °C to remove debris, and the supernatants were collected for analysis.

Protein concentration in cell lysates was determined using the Bradford assay, based on the binding of Coomassie Brilliant Blue G-250 (Sigma-Aldrich; St. Louis, MO, USA) dye to proteins [21]. Upon binding primarily to basic and aromatic amino acid residues, the dye undergoes a spectral shift from its red form (absorbance maximum at 465 nm) to a blue form with a maximum at 595 nm, which is proportional to the protein concentration. Briefly, cells were lysed using an appropriate lysis buffer, and the lysates were clarified by centrifugation. Aliquots of the supernatants were mixed with Bradford reagent (200 µL of reagent with 10 µL of sample or standard) and incubated at room temperature for 5–10 min. A standard curve was prepared using bovine serum albumin (BSA) at known concentrations. Absorbance was measured at 595 nm using a microplate reader. Protein concentrations in the samples were calculated from the standard curve.

Melanocytes and SK-Mel-5 cells were divided into eight experimental groups, each comprising five independent samples:

Control—melanocytes or SK-Mel-5 cells cultured in standard medium containing;

0.3% ethanol as a vehicle control;

CBD—cells treated with CBD (5 µM) for 24 h;

CBG—cells treated with CBG (1 µM) for 24 h;

CBG + CBD—cells treated with CBD (5 µM) and CBG (1 µM) for 24 h;

UVA—cells exposed to UVA radiation (18 J/cm^2^) and subsequently cultured for 24 h in standard medium (0.3% ethanol);

UVA + CBD—cells exposed to UVA and then treated with CBD (5 µM) for 24 h;

UVA + CBG—cells exposed to UVA and then treated with CBG (1 µM) for 24 h;

UVA + CBG + CBD—cells exposed to UVA and then treated with CBD (5 µM) and CBG (1 µM) for 24 h.

### 2.3. Assessment of Cell Viability, Proliferation, and Cell Death Ratio

For cell viability measurement made by MTT test, melanocytes and SK-Mel-5 cells were seeded and treated in 96-well plates. Cell seeding density was 10^4^ cells per well. Non-irradiated cells, as well as cells following their exposure to UVA radiation (18 J/cm^2^), were incubated for 24 h in a medium supplemented with CBD (5 µM), CBG (1 µM), or their combination (5 µM CBD + 1 µM CBG). To prevent solvent-induced effects, all cell media contained the 0.3% ethanol. Measurements for each experimental group were performed in 5 independent wells. Following 24 h incubation, medium from each well was removed, and cells were incubated with 0.25 mg/mL MTT solution in PBS for 40 min at a temperature of 37 °C. MTT was removed, and cells were lysed using dimethyl sulfoxide. The absorbance of formed formazan was read at 570 nm [20] using a Multiskan GO Microplate Spectrophotometer (Thermo Fisher Scientific; Waltham, MA, USA). Background absorbance was corrected by subtracting the absorbance of wells that contain only culture medium, without seeded cells, from all experimental well readings. Results were presented as a percentage of the value obtained for control cells.

To perform sulforhodamine B (SRB)-based proliferation assay, cells were seeded in 96-well plates. Measurements for each experimental group were performed in 5 independent wells. Following incubation, cells were washed with warm PBS (37 °C) and fixed with 10% trichloroacetic for 5 min. Next, cells were incubated with 0.4% (*w*/*v*) SRB solution in 10% acetic acid for 30 min. Extensive five times washes with distilled water was applied. The bond SRB was extracted with 10 mM Tris-base solution. The absorbance was read at 540 nm [22] using a Multiskan GO Microplate Spectrophotometer (Thermo Fisher Scientific; Waltham, MA, USA). Results were presented as a percentage of the value obtained for control cells.

For staining with trypan blue dye, cells were seeded in 24-well plates [23]. Measurements for each experimental group were performed in 5 independent wells. Following incubation, cells were washed with warm PBS (37 °C), scraped and suspended in this buffer. Next, 10 µL of cell suspension was mixed with 10 µL of 0.4% trypan blue solution. After 3 min incubation 10 µL of mixture was loaded onto a hemocytometer. Under a light microscope (NikonEclipse Ti microscope combined with MagingSource camera operated by Nikon’s NIS-Elements imaging software version 5.30.02 (Nikon Instruments Inc., Melville, NY, USA)) the number of stained dead cells for every 100 cells were counted. Results were presented as a percentage of the death cells in each experimental culture. The detailed results for all assays are provided in Supplementary File S2.

### 2.4. Determination of Pro-Oxidant Parameters

Total ROS generation was measured using an electron spin resonance (ESR) spectrometer e-scan (Noxygen GmbH/Bruker Biospin GmbH, (Elzach, Germany) [24]. Cell suspensions were resuspended in freshly prepared Krebs/HEPES buffer (pH 7.4). For ROS detection, 96 µL of cell suspension was incubated with the cell-permeable spin probe CMH (1-hydroxy-3-methoxycarbonyl-2,2,5,5-tetramethylpyrrolidine; final concentration 400 µM; in the presence of metal chelators: deferoxamine methanesulfonate salt (DF; 25 µM) and sodium diethyldithiocarbamate trihydrate (DETC; 5 µM). These additives were included to prevent metal-catalyzed auto-oxidation and to stabilize radical intermediates. Samples were incubated for 30 min at 37 °C under controlled conditions (protected from light to avoid photo-oxidation). During incubation, ROS oxidize CMH to the stable nitroxide radical 3-methoxycarbonyl-proxyl (CM•), which accumulates proportionally to total ROS production. After incubation, samples were immediately transferred into ESR capillary tubes, avoiding air bubbles, and analyzed without delay to minimize signal decay. ESR measurements were performed using the following typical instrument settings: center field ~3480 G, sweep width ~60 G, microwave frequency ~9.75 GHz, microwave power ~20 mW, modulation amplitude ~2 G, and sweep time ~30 s. Signal intensity was quantified as the first derivative of the ESR spectrum and expressed as peak-to-peak amplitude. Quantification of ROS production was achieved by comparison with an external standard curve generated using known concentrations of the CM radical. Finally, ROS generation was expressed as µM CM formed per minute per mg of protein (µM/min/mg protein).

NADPH oxidase (NOX; EC 1.6.3.1) activity was assessed using a protocol adapted from Griendling et al. [25]. For the NOX activity assay, 50 µg of total protein (in 10 µL of lysate) was added to 90 µL of reaction mixture containing 50 mM phosphate buffer (pH 7.0), 1 mM EGTA, 150 mM sucrose, 400 µM NADPH (electron donor), and 250 µM lucigenin as a chemiluminescent probe. The reaction was initiated by the addition of NADPH, and chemiluminescence was immediately measured using a microplate luminometer under dark conditions. Luminescence signals were recorded at 1 min intervals over a period of 10 min at 37 °C. Background luminescence (measured in samples without NADPH or in buffer alone) was subtracted from all readings. NOX activity was calculated from the linear increase in luminescence over time and expressed as milli-relative light units per minute per milligram of protein (mRLU/min/mg protein).

Xanthine oxidase (XO; EC 1.17.3.2) activity was determined by measuring uric acid (UA) formation from xanthine, as previously described [26]. XO activity was assayed spectrophotometrically by monitoring the formation of uric acid from xanthine. The reaction mixture contained 50 mM phosphate buffer (pH 7.4) and xanthine (0.5 mM) as a substrate. The reaction was initiated by the addition of 10 µL cell lysate and carried out at 37 °C. The increase in absorbance at 290 nm, corresponding to uric acid formation, was recorded continuously over time using a UV–Vis spectrophotometer. Measurements were performed in quartz cuvettes to ensure accurate detection in the UV range. A blank sample, containing all reaction components except xanthine, was run in parallel to account for background absorbance. XO activity was calculated as the difference between the rate of absorbance increase in the complete reaction mixture and the blank. The concentration of uric acid formed was determined using its molar extinction coefficient (ε = 12.2 mM^−1^·cm^−1^ at 290 nm), and enzyme activity was expressed as the amount of enzyme catalyzing the formation of 1 µmol of uric acid per minute. Finally, XO activity was normalized to protein content and expressed as milliunits per milligram of protein (mU/mg protein).

### 2.5. Determination of Antioxidant Parameters

The expression levels of Nrf2 i HO-1 were measured using an ELISA method [27]. Cell lysates diluted two times in 0.05 M sodium carbonate (pH 9.6) were applied to pre-coated with primary anti-Nrf2 and anti-HO-1 antibodies ELISA plate wells (Nunc Immuno Maxi Sorp, Thermo Scientific, Waltham, MA, USA) and incubated at 4 °C for 5 h to link with plate. Antibodies against heme oxygenase 1 (HO-1) (host: mouse), and phospho-Nrf2 (Ser40) (host: rabbit) were used at a dilution of 1:1000 in 1% bovine serum albumin in PBS. After washing with PBS supplemented with 0.1% Tween 20, samples were incubated 3 h with a blocking solution (5% non-fat dry milk in carbonate binding buffer) and then incubated overnight at 4 °C with primary antibodies against Nrf2 and HO-1. Following washing, plates were incubated for 30 min with peroxidase blocking solution (3% H_2_O_2_, 3% non-fat dry milk in PBS) at room temperature. EnVision+ Dual Link System-HRP (diluted 1:100) against rabbit/mouse was used. After 1 h of incubation at room temperature, the secondary anti-body was removed, and the plates were incubated with chromogen substrate solution (0.1 mg/mL TMB, 0.012% H_2_O_2_) for 40 min. The reaction was stopped by 2 M sulfuric acid, and absorbance was measured at 450 nm within 10 min. Results were calculated using standard curves generated for each protein HO-1 (0.1–5 ng/mg protein) and phospho-Nrf2 (Ser40) (0.1–5 ng/mg protein).

The activities of superoxide dismutase isoforms, cytosolic Cu/Zn-dependent SOD (SOD1) and mitochondrial Mn-dependent SOD (SOD2) (EC 1.15.1.1), were determined using a spectrophotometric method based on the inhibition of adrenaline auto-oxidation to adrenochrome, as previously described [28,29]. To differentiate between SOD isoforms, aliquots of the lysate were treated with 2% sodium dodecyl sulfate (SDS) and incubated at 37 °C for 30 min to selectively inactivate Mn-SOD (SOD2), while preserving the activity of Cu,Zn-SOD (SOD1). Total SOD activity (SOD1 + SOD2) was measured in untreated samples, whereas SOD1 activity was determined in SDS-treated samples. SOD2 activity was calculated as the difference between total SOD and SOD1 activity. The assay was performed in alkaline conditions (pH 10.2) using carbonate buffer (50 mM, containing 0.1 mM EDTA). The reaction mixture consisted of appropriately diluted cell lysate and freshly prepared adrenaline (epinephrine) solution (0.3 mM), which undergoes spontaneous oxidation to adrenochrome in the presence of superoxide anions. The increase in absorbance at 480 nm, corresponding to adrenochrome formation, was monitored spectrophotometrically at 25 °C for 10 min. One unit of SOD activity was defined as the amount of enzyme required to inhibit the rate of adrenaline oxidation by 50% under assay conditions. Final results were expressed as units per milligram of protein (U/mg protein).

Glutathione peroxidase (GPx; EC 1.11.1.6) activity was determined using a coupled spectrophotometric assay according to the method of Paglia and Valentine [30], using the method based on the reduction of organic peroxides in the presence of NADPH. The GPx activity assay was performed in a reaction mixture containing 50 mM phosphate buffer (pH 7.0), 1 mM EDTA, reduced glutathione (1 mM), NADPH (0.2 mM) and an organic peroxide substrate (hydrogen peroxide, 0.25 mM). The reaction was initiated by the addition of the peroxide substrate. In this coupled system, GPx catalyzes the reduction of the peroxide at the expense of GSH, forming oxidized glutathione (GSSG), which is subsequently reduced back to GSH by glutathione reductase with concomitant oxidation of NADPH to NADP^+^. The decrease in NADPH absorbance was monitored continuously at 340 nm using a UV/Vis spectrophotometer at 25 °C. The rate of NADPH oxidation, reflected by the decrease in absorbance at 340 nm, was proportional to GPx activity. Calculations were performed using the molar extinction coefficient for NADPH (ε = 6.22 mM^−1^·cm^−1^). One unit of GPx activity was defined as the amount of enzyme required to oxidize 1 µmol of NADPH per minute under assay conditions. Enzyme activity was normalized to protein content and expressed as milliunits per milligram of protein (mU/mg protein).

Glutathione reductase (GR; EC 1.6.4.2) activity was determined using a spectrophotometric method based on the NADPH-dependent reduction of oxidized glutathione (GSSG), as previously described [31], The GR activity assay was performed in a reaction mixture containing 100 mM phosphate buffer (pH 7.1), 1 mM EDTA, oxidized glutathione (1.0 mM), KCl (0.2 mM) and NADPH (0.2 mM). The reaction was initiated by the addition of 10 µL cell lysate. GR catalyzes the reduction of GSSG to reduced glutathione (GSH) using NADPH as an electron donor, resulting in the oxidation of NADPH to NADP^+^. The decrease in NADPH absorbance was monitored continuously at 340 nm using a UV–Vis spectrophotometer at 25 °C. The rate of absorbance decrease at 340 nm was directly proportional to GR activity and was calculated using the molar extinction coefficient of NADPH (ε = 6.22 mM^−1^·cm^−1^). One unit of GR activity was defined as the amount of enzyme required to oxidize 1 mmol of NADPH per minute under assay conditions. Enzyme activity was normalized to protein content and expressed as milliunits per milligram of protein (mU/mg protein).

The intracellular level of reduced glutathione (GSH) was determined using capillary electrophoresis (CE) according to Nuria Maeso et al. (2005) [32]. In accordance with the original method, GSH was separated without prior derivatization using capillary electrophoresis. 100 µL of cell lysate was deproteinized using acetonitrile (ACN), followed by centrifugation. The resulting supernatant was collected and subjected to further analysis. Analyses were performed on a fused-silica capillary (effective length 40 cm, internal diameter 50 µm). The separation was carried out under normal polarity at a constant voltage of 27 kV. The running buffer consisted of a borate-based electrolyte (50 mM sodium borate, pH ~9.0), which enables efficient separation of low-molecular-weight thiols. Capillary temperature was maintained at 25 °C to ensure reproducibility of migration times. Samples were introduced hydrodynamically, and between runs, the capillary was conditioned by sequential rinsing with NaOH, water, and running buffer to maintain separation efficiency. Detection of GSH was performed using a UV detector set at 200 nm, based on the intrinsic absorbance of the peptide bond. Peak identification was achieved by comparing migration times with those of authentic GSH standards analyzed under identical conditions. Quantification was performed using external calibration curves constructed with known concentrations of reduced glutathione. Final results were expressed as nmol of GSH per mg of protein (nmol/mg protein).

Thioredoxin reductase (TrxR; EC 1.8.1.9) activity was determined using a commercially available colorimetric assay kit from Sigma-Aldrich, according to the manufacturer’s instructions and as previously described [33]. The assay is based on the ability of TrxR to catalyze the NADPH-dependent reduction of 5,5′-dithiobis(2-nitrobenzoic acid) (DTNB) to 5-thio-2-nitrobenzoic acid (TNB), a yellow-colored product. The reaction mixture contained NADPH as an electron donor and DTNB as a chromogenic substrate. Upon reduction, TNB formation was monitored spectrophotometrically by measuring the increase in absorbance at 412 nm. The reaction was initiated by the addition of cell lysate and carried out at 25 °C. The rate of increase in absorbance at 412 nm was recorded. Blank samples (without enzyme or without NADPH) were included to correct for non-enzymatic reduction of DTNB. TrxR activity was calculated based on the rate of TNB formation using its molar extinction coefficient (ε = 13.6 mM^−1^·cm^−1^). One unit of TrxR activity was defined as the amount of enzyme catalyzing the formation of 1 µmol of TNB per minute at 25 °C. Enzyme activity was normalized to total protein content and expressed as units per milligram of protein (U/mg protein).

Thioredoxin (Trx) level was quantified using ELISA method [27], as described above. Anti-thioredoxin antibody (Abcam, Cambridge, MA, USA) (host: rabbit) was used as the primary antibody, and goat anti-rabbit/mouse EnVision+ Dual Link/HRP solution (1:100) was used as the secondary antibody. Thioredoxin concentration was calculated using standard curve (0.05–2 µg/mg protein).

### 2.6. Assessment of Pro-Inflammatory Proteins

The expression levels of selected pro-inflammatory non-enzymatic proteins were measured using an ELISA method [27], as described above. Antibodies against NF-κB (p65), NF-κB (p52), tumor necrosis factor-alpha (TNF-α) (host: mouse) were used as the primary antibody, and goat anti-rabbit/mouse EnVision+ Dual Link/HRP solution (1:100) was used as the secondary antibody. Results were calculated using standard curves generated for each protein (NF-κB (p65) (0.02–1 ng/mg protein), TNF-α (0.1–3 ng/mg protein) and NF-κB (p52) (0.2–5 ng/mg protein).

### 2.7. Examination of Lipid Metabolism Products

The level of 4-hydroxy-2-nonenal (4-HNE) was analyzed by gas chromatography coupled with mass spectrometry (7890A GC–7000 triple quadrupole MS, Agilent Technologies, Palo Alto, CA, USA) according to Luo’s method [34]. First, 4-HNE was derivatized into the corresponding PFB oximes using PFB-hydroxylamine as the derivatizing reagent. Benzaldehyde-d6 was added to the samples as an internal standard. After incubation with methanol, the samples were deproteinized and subsequently extracted with hexane. The hexane layer was evaporated, and N,O-bis(trimethylsilyl)trifluoroacetamide containing 1% trimethylchlorosilane was added. Aliquots of 1 µL were then loaded onto the chromatographic column, which consisted of a fused-silica HP-5 ms capillary column (30 m × 0.25 mm; (Agilent Technologies; Santa Clara, CA, USA). Analyte levels were measured in selected ion monitoring (SIM) mode. The ions monitored for the PFB 4-HNE oxime and benzaldehyde-d6 were *m*/*z* 333.0, 181.0, and 245.0. 4-HNE levels were expressed as nmol/µg protein.

The level of F2-isoprostanes, expressed as 8-isoPGF2α, was quantified using a modified method of Coolen et al. [35] with high-performance liquid chromatography coupled to a triple quadrupole mass spectrometer (1290 Infinity II HPLC-6460 triple quadrupole MS, Agilent Technologies, Palo Alto, CA, USA). After adding the internal standard (8-iso-PGF2α-d4), the samples were acidified to pH 3 and centrifuged. 8-isoPGF2α was then extracted by solid-phase extraction (SPE). Ionization was performed using negative electrospray ionization (ESI) in multiple reaction monitoring (MRM) mode. Quantification was performed by monitoring the transitions from m/z 353.2 to 193.1 for 8-isoPGF2α and from *m*/*z* 357.2 to 197.1 for the internal standard. F2-isoprostanes levels were expressed as ng/mg protein.

### 2.8. Determination of 4-HNE-Protein Adducts

Protein modification by lipid peroxidation products was assessed by measuring 4-HNE-protein adducts using ELISA [27] as previously described. A monoclonal anti-4-HNE-protein mouse antibody (Invitrogen, Burlington, ON, Canada) was used as the primary antibody, and goat anti-rabbit/mouse EnVision+ Dual Link/HRP solution (1:100) (Agilent Technologies, Santa Clara, CA, USA) was used as the secondary antibody. 4-HNE-protein adducts concentration was calculated using standard curve prepared by incubating 4-HNE with BSA at increasing concentrations (0.05–2 µg/mg protein).

### 2.9. Statistical Analysis

Data were assessed for normality using the Shapiro–Wilk test and were presented as mean ± standard deviation (SD) from five independent biological replicates (*n* = 5) in three technical replicates for each parameter. Statistical analysis was performed using two-way ANOVA followed by Tukey’s post hoc test to evaluate differences between groups and interactions between factors. All experiments were conducted on cell lines, with technical replicates used for each experiment, which do not reflect biological variability. A *p*-value < 0.05 was considered statistically significant.

## 3. Results

### 3.1. Prooxidative Parameters

**Figure 2:**
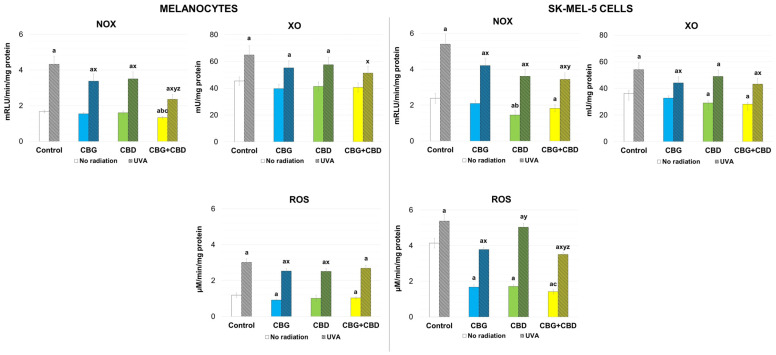
The activity of pro-oxidative enzymes (NOX, XO) and ROS level in the melanocytes and SK-Mel-5 cells. The mean ± SD values (*n* = 5) are presented with statistically significant differences: a—vs. control group; b—vs. CBG group; c—vs. CBD group; x—vs. UVA group; y—vs. UVA + CBG group; z—vs. UVA + CBD group; *p* < 0.05.

The results obtained in this study indicate that CBG and CBD, in used concentrations, either individually or in combination, did not cause changes in the morphology or viability of non-irradiated melanocytes and SK-Mel-5 cells (Figures S1 and S2), but had a significant impact on the function of both non-irradiated and UVA-irradiated cells. This was evident in the regulation of redox balance, as reflected by the reduced activity of enzymes responsible for ROS generation and, consequently, lower total ROS production (Figure 2). In unirradiated melanocytes, application of phytocannabinoids alone had no significant effect on NOX and XO activity, whereas combined CBG and CBD reduced NOX activity by ~20% and ROS levels by ~15%. Furthermore, CBG reduced ROS levels, comparable to the effects observed with both phytocannabinoids combined. However, in SK-Mel-5 cells, CBD and phytocannabinoids combination decreased NOX activity by ~40%, and ~24%, respectively, while XO activity was reduced by ~20% following CBD and combined treatment. ROS levels were lowered in all treated groups, with the strongest reduction (~60%) observed after combined treatment. UVA irradiation increased NOX activity and ROS levels by ~2-fold in melanocytes, and NOX activity by 2-fold in SK-Mel-5 cells. These changes were accompanied by a significant decrease in cell viability (Figure S2). Moreover, ROS levels were also increased in SK-Mel-5 cells, but to a much lesser extent than in melanocytes. In both cell types, UVA exposure increases XO activity by approximately 30%. In irradiated melanocytes, CBG and CBD reduced NOX activity and ROS levels by ~20–30%. Moreover, treatment with CBG + CBD reduces NOX activity by approximately 2-fold and XO activity by about 21%. In SK-Mel-5 cells, stronger effects were observed, with reductions of ~25–40% in NOX activity, ~30–35% in XO activity, and ~30% in ROS levels. These changes were accompanied by decreased oxidative stress and cell damage in both melanocytes and SK-Mel-5 cells, suggesting a role in preventing cell death (Figure S4).

### 3.2. Antioxidative Parameters

**Figure 3:**
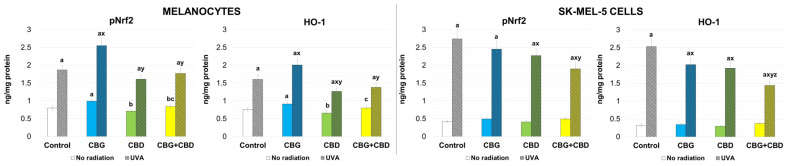
The level of pNrf2 and HO-1 in the melanocytes and SK-Mel-5 cells. The mean ± SD values (*n* = 5) are presented with statistically significant differences: a—vs. control group; b—vs. CBG group; c—vs. CBD group; x—vs. UVA radiation group; y—vs. UVA + CBG group; z—vs. UVA + CBD group; *p* < 0.05.

CBG and CBD modulated antioxidant responses at both the transcriptional (pNrf2) and protein (HO-1) levels in melanocytes and SK-Mel-5 cells (Figure 3). In non-irradiated melanocytes, CBG increased pNrf2 and HO-1 levels by approximately 20%. In SK-Mel-5 cells, however, phytocannabinoids did not increase p-Nrf2 and HO-1 expression. UVA irradiation markedly increased pNrf2 (~2–3-fold) and HO-1 (~2-fold) in melanocytes, and a greater extent in SK-Mel-5 cells (7-fold). In irradiated melanocytes, CBG further increased pNrf2 and HO-1 levels by ~25–40% compared to UVA alone. Notably, HO-1 levels were significantly decreased following CBD treatment (~20%). In UVA-irradiated SK-Mel-5 cells, phytocannabinoid treatment led to a consistent reduction in both pNrf2 and HO-1 levels. CBG, CBD, and combined treatment decreased pNrf2 by ~10–30% and HO-1 by ~20–40% relative to UVA-exposed cells. Importantly, none of the treatments restored pNrf2 or HO-1 levels to control values in either cell type.

**Figure 4:**
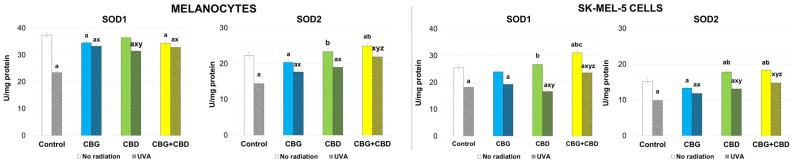
Activity of SOD1 and SOD2 in the melanocytes and SK-Mel-5 cells. The mean ± SD values (*n* = 5) are presented with statistically significant differences: a—vs. control group; b—vs. CBG group; c—vs. CBD group; x—vs. UVA radiation group; y—vs. UVA + CBG group; z—vs. UVA + CBD group; *p* < 0.05.

CBG and CBD differentially modulated SOD1 and SOD2 activity in melanocytes and SK-Mel-5 cells (Figure 4). In non-irradiated melanocytes, CBG reduced SOD1 and SOD2 activity by ~10–20%. The combined treatment slightly decreased SOD1 activity (~5–10%) but increased SOD2 by ~10–20% compared to control. In SK-Mel-5 cells, CBG revealed minimal effect on SOD1 but reduced SOD2 (~10–15%), whereas CBD increased SOD2 activity (~15–20%). The combined treatment increased SOD1 (~20–25%) and SOD2 activity (~20–25%). However, UVA irradiation decreased SOD1 and SOD2 activity by ~30–40% in melanocytes and by ~20–30% in SK-Mel-5 cells. In irradiated melanocytes, CBG, CBD, and CBG + CBD increased activity of SOD1 by ~30–50% and SOD2 by ~20–40% relative to UVA-treated cells. In UVA-irradiated SK-Mel-5 cells, CBG had no significant effect on SOD1, CBD reduced it by ~10–20%, while the combined treatment increased it by ~20–30%. In contrast, SOD2 activity was increased by all treatments (~20–40%), with the strongest effect observed for the combined CBG + CBD treatment.

**Figure 5:**
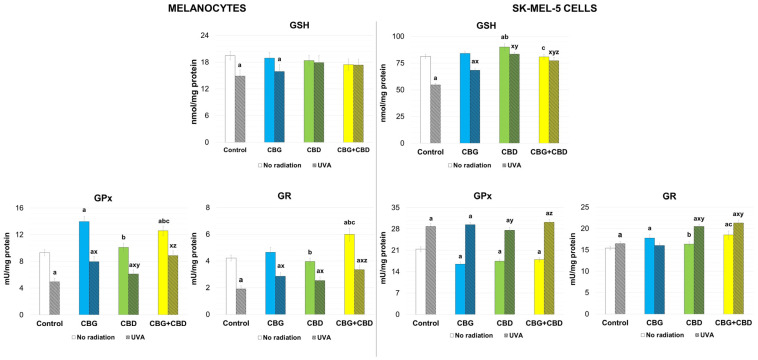
The level of GSH and the activity of enzymes (GPx, GR) in the melanocytes and SK-Mel-5 cells. The mean ± SD values (*n* = 5) are presented with statistically significant differences: a—vs. control group; b—vs. CBG group; c—vs. CBD group; x—vs. UVA radiation group; y—vs. UVA + CBG group; z—vs. UVA + CBD group; *p* < 0.05.

Phytocannabinoids also modulate the efficiency of the glutathione system components in both melanocytes and SK-Mel-5 cells (Figure 5). In non-irradiated melanocytes, CBG increased GPx activity by ~50%. The combined CBG + CBD treatment elevated GPx by ~35% and GR activity by ~30%. GSH levels remained largely unchanged across treatments. However, in SK-Mel-5 cells, CBD increased GSH levels by ~10%, while CBG and CBG + CBD had minimal effects. All treatments decreased GPx activity by ~15–25%. In contrast, GR activity increased by ~16% following CBG and by ~20% after combined treatment. UVA irradiation reduced GSH levels in melanocytes by ~24% and decreased GPx and GR activity approximately 2-fold. However, in SK-Mel-5 cells, UVA reduced GSH by ~33%, while increasing GPx and GR activity by ~10–35%. In UVA-irradiated melanocytes, all phytocannabinoid treatments increased GPx activity by ~24–80% and GR activity by ~25–75% relative to UVA-exposed cells. GSH levels were partially restored; however, the change was not statistically significant. In UVA-irradiated SK-Mel-5 cells, CBD and CBG + CBD restored GSH levels to near control values (~40–50% increase vs. UVA). CBG also increases GSH levels, but to a lesser extent by ~25%. GPx activity remained largely unchanged compared to UVA conditions, whereas GR activity increased by ~20–30%, particularly after CBD and combined treatment.

**Figure 6:**
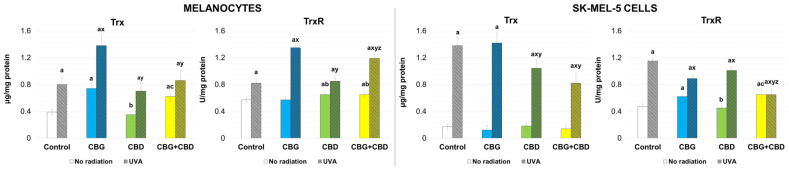
The level of Trx and the activity of TrxR in the melanocytes and SK-Mel-5 cells. The mean ± SD values (*n* = 5) are presented with statistically significant differences: a—vs. control group; b—vs. CBG group; c—vs. CBD group; x—vs. UVA radiation group; y—vs. UVA + CBG group; z—vs. UVA + CBD group; *p* < 0.05.

The second system involved in the protection of membrane phospholipids, the thioredoxin system, also changed under the influence of used chemical and physical factors (Figure 6). In non-irradiated melanocytes, CBG increased Trx levels approximately 2-fold, while CBG + CBD elevated them by ~60%. TrxR activity increased following CBD and CBG + CBD treatment (~14%), whereas CBG had no effect. In SK-Mel-5 cells, phytocannabinoids variably affected Trx levels; however, none of these changes reached statistical significance. TrxR activity increased after CBG (~32%), whereas combined treatment resulted in a moderate increase (~38%). UVA irradiation increased Trx levels by ~2-fold in melanocytes and by ~8-fold in SK-Mel-5 cells. TrxR activity increased by ~44% in melanocytes and ~2.5-fold in SK-Mel-5 cells. In UVA-irradiated melanocytes, CBG further increased Trx levels by ~73%. TrxR activity increased following CBG (~65%) and CBG + CBD (~45%), whereas CBD alone revealed a weaker effect. In UVA-irradiated SK-Mel-5 cells, phytocannabinoids reduced Trx levels, with decreases of ~25% for CBD and ~40% for CBG + CBD. In contrast to melanocytes, TrxR activity was reduced by all treatments (~12–43%) compared to UVA-exposed cells.

### 3.3. Lipid Peroxidation Markers

**Figure 7:**
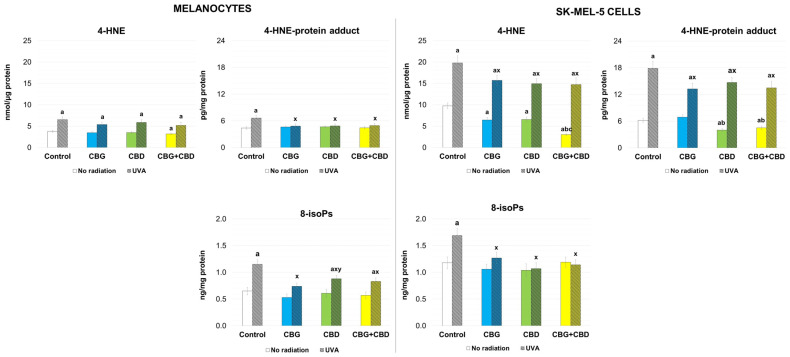
The level of biomarkers of lipid peroxidation (4-HNE, 4-HNE-protein adducts, 8-isoPs) in the melanocytes and SK-Mel-5 cells. The mean ± SD values (*n* = 5) are presented with statistically significant differences: a—vs. control group; b—vs. CBG group; c—vs. CBD group; x—vs. UVA radiation group; y—vs. UVA + CBG group; *p* < 0.05.

Disturbances in redox balance are accompanied by changes in the levels of oxidative modification products of lipids and proteins (Figure 7). In melanocytes, phytocannabinoids under non-irradiated conditions had minimal effects on the level of 4-HNE and 4-HNE–protein adducts, while CBG slightly reducing 8-isoPs levels (~20%). In SK-Mel-5 cells, CBG and CBD reduced 4-HNE levels by ~35%, with the strongest decrease observed after combined treatment (~70%). Similar changes were observed for 4-HNE-protein adducts, particularly in response to CBD and CBG + CBD treatment (~27–35% reduction). Under control conditions, phytocannabinoids did not affect the level of the analyzed isoprostane. UVA irradiation increased 4-HNE levels by ~73% in melanocytes and ~2-fold in SK-Mel-5 cells, and 4-HNE-protein adduct levels increased by ~50% in melanocytes and about 3-fold in SK-Mel-5 cells. However, 8-isoPs levels increased by ~77% and ~43%, respectively. In UVA-irradiated SK-Mel-5 cells treated with phytocannabinoids, it had a limited effect on 4-HNE, while the level of 4-HNE-protein adducts returned to levels similar to control, while the concentration of 8-isoPs decreased by ~23–36% compared with the UVA group. In UVA-irradiated SK-Mel-5 cells, phytocannabinoids reduced the concentration of 4-HNE by ~20–25% and the concentration of 4-HNE-protein adducts by ~18–26%, although both parameters remained elevated compared with the control group. In contrast, the level of 8-isoPs was reduced by ~25–37% after phytocannabinoid treatment, approaching control values.

### 3.4. Proinflammatory Parameters

**Figure 8:**
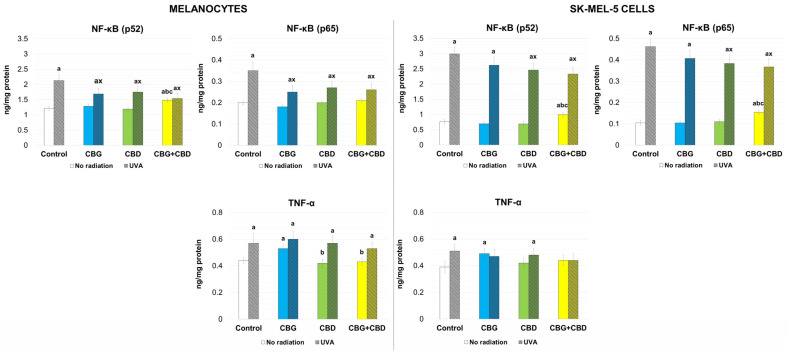
The level of NF-κB subunits (p52, p65) and TNF-α in the melanocytes and SK-Mel-5 cells. The mean ± SD values (*n* = 5) are presented with statistically significant differences: a—vs. control group; b—vs. CBG group; c—vs. CBD group; x—vs. UVA radiation group; *p* < 0.05.

Both UVA radiation and phytocannabinoids modulate cellular reactions at the level of redox balance and also influence the pro-inflammatory response of cells, which is reflected in the altered expression of the cellular pro-inflammatory transcription factor NF-κB (Figure 8). In control melanocytes exposed to UVA radiation, CBG treatment alone increased TNF-α levels by ~20%, whereas combined CBG and CBD treatment increased the p52 subunit of NF-κB by ~22%. In UVA-exposed SK-Mel-5 cells, CBG also increased TNF-α levels by ~26%, whereas combined CBG and CBD treatment alone increased the levels of NF-κB subunits, and p52 and p65 levels increased by ~30% and ~47%, respectively. In contrast, exposure of melanocytes to UVA radiation resulted in an increase in the level of NF-κB subunits in melanocytes (p52: ~2-fold; p65: ~75%) and in SK-Mel-5 cells (p52: ~4-fold; p65: ~4.5-fold), along with an increased level of TNF-α in melanocytes, as well as in SK-Mel-5 cells by ~30%. In UVA-irradiated melanocytes, phytocannabinoids attenuated this response, reducing the level of NF-κB subunits (p52: ~18–28%; p65: ~25–29%). A similar trend was observed in SK-Mel-5 cells, where phytocannabinoids reduced the level of NF-κB subunits (p52: −20%; p65: −20%), although the effect of CBG was not statistically significant. It is worth noting that none of the phytocannabinoids tested caused a reduction in TNF-α levels.

## 4. Discussion

### 4.1. Comparison of the Effects of UVA Radiation on Redox Balance and Inflammatory Signaling in Melanocytes and SK-Mel-5 Cells

UVA radiation, which accounts for approximately 95% of the ultraviolet radiation reaching the Earth’s surface, plays a key role in skin photobiology and photocarcinogenesis [36,37]. Therefore, human skin is exposed to UVA radiation daily at varying doses, depending on climate, season, and lifestyle. In temperate climates, it ranges from 10 to 40 J/cm^2^. Consequently, the dose of 18 J/cm^2^ used in the experiment corresponds to the average exposure and UVA doses used in phototherapy for skin diseases such as psoriasis and vitiligo [38,39]. Such UVA doses generate many benefits for the human body, including an increase in neuroendocrine mediators in the blood (e.g., melatonin) and vitamin D biosynthesis [40,41]. However, excessive exposure of melanocytes to UVA radiation promotes prolonged melanin biosynthesis and activation of endogenous chromophores, resulting in excessive ROS production, accompanied by increased activity of pro-oxidant enzymes such as NOX and XO [42], which may shift melanin biosynthesis towards pheomelanogenesis, further increasing ROS generation and promoting the development of carcinogenic lesions, particularly in dysplastic nevi [43]. Consequently, the overproduction of ROS observed in both melanocytes and melanoma cells exposed to UVA and the accompanying oxidative stress may play a complex role [44]. It is known that elevated ROS levels can promote both cancer initiation and progression by inducing mutations and activating pro-oncogenic signaling pathways. At the same time, however, melanoma cells develop adaptive mechanisms that enable them to tolerate increased oxidative stress [44]. UVA-induced ROS modulate mitogen-activated protein kinase (MAPK), NF-κB, and p53 pathways involved in melanoma development and therapy resistance [45,46]. Therefore, understanding the consequences of UVA-induced oxidative stress in both melanocytes and melanoma cells is believed to be important for elucidating the pathogenesis of melanoma and may contribute to the development of new preventive strategies and targeted therapies [36,44].

The present results indicate that although UVA also causes increased NOX and XO activity in melanoma cells, the increase in ROS levels is less pronounced than in melanocytes. However, it is known that moderately elevated ROS levels can support proliferation, invasion, angiogenesis, and metastasis in melanoma cells, whereas overproduction of ROS can induce apoptosis and cell senescence [47,48]. These processes are primarily mediated by the MAPK, phosphoinositide 3-kinase (PI3K)/protein kinase B (PKB), AKT/mTOR (mammalian target of rapamycin), and NF-κB signaling pathways [8]. To counteract elevated ROS levels, cells significantly stimulate the Nrf2-dependent antioxidant pathway, which is supported by the present results showing significant upregulation of Nrf2 and its target protein HO-1 observed in SK-MEL-5 cells. It is known, however, that increased Nrf2 expression is frequently observed in cancer cells and contributes to increased antioxidant biosynthesis, including increased expression of antioxidant enzymes [49]. In addition to regulating redox balance, Nrf2 is also considered a proto-oncogenic factor, whose increased expression has been documented in several malignancies, including colorectal cancer, breast cancer, and melanoma [50,51,52]. However, increased nuclear expression of Nrf2 in melanoma patients has previously been shown to correlate with poorer survival outcomes [53], while in vitro studies have shown that Nrf2 activation weakens the innate immune response in melanoma cells [54], which is associated with increased levels of lipid peroxidation products, including 4-HNE, a recognized signaling molecule [55]. It is known, however, that an increase in 4-HNE levels following exposure to UVA radiation by modifying the structure of the Nrf2 inhibitor, Keap1, increases the transcriptional efficiency of Nrf2 [56]. Consequently, melanoma cells become more resistant to external factors, including anticancer therapies. Furthermore, increased Nrf2 efficiency may protect melanoma cells against ferroptosis [57,58].

However, despite elevated Nrf2 levels, UVA exposure reduces the activity of key antioxidant enzymes, including SODs, and lowers GSH levels in both melanocytes and SK-MEL-5 cells, thereby shifting the redox balance towards oxidation. As a result, both normal and cancer cells show marked changes in the activity of GSH-dependent enzymes. In melanocytes, GSH deficiency reduces the antioxidant capacity of GPx and GR, thereby exacerbating the redox imbalance [59], whereas in melanoma cells, reduced GSH levels can stimulate further hyperproliferation of cancer cells [60]. A similar phenomenon is observed in the Trx-dependent system, which is strongly activated in UVA-irradiated SK-MEL-5 cells as part of cellular adaptation to carcinogenesis. However, it is known that depletion of endogenous GSH and reduced TrxR activity in SK-MEL-5 cells inhibit tumor growth, potentially creating opportunities for effective anticancer therapy [61,62].

Consequently, UVA-induced oxidative stress leads to damage not only to proteins but also to lipids, as evidenced by increased levels of the lipid oxidation product 4-HNE, and consequently, further modifications of protein structure-efficiency due to the formation of 4-HNE-protein adducts. The resulting redox imbalance may promote melanogenesis, proliferation of precancerous melanocytes, and their transformation into melanoma, partly through activation of the NF-κB, Sesn2, and AKT pathways [63,64]. This may be related to the fact that 4-HNE-protein adducts induce proinflammatory signaling, including activation of the NF-κB/TNF-α pathway [65], which is observed after UVA irradiation of both melanocytes and melanoma cells, although this effect is more pronounced in SK-MEL-5 cells. In contrast, increased expression of TNF-α increases matrix metalloproteinase (MMP) activity, thereby promoting melanoma cell proliferation, migration, and metastasis [66].

These results suggest that UVA-induced oxidative stress can induce a broad antioxidant response, particularly in SK-MEL-5 cells, but may also contribute to the neoplastic transformation of melanocytes. Simultaneously, increased NF-κB-dependent proinflammatory signaling may indicate the possibility of multifaceted metabolic changes in cells, which may exacerbate other pro-carcinogenic effects. Consequently, this supports the ongoing search for compounds with protective and therapeutic effects.

### 4.2. Effect of Phytocannabinoids on the Redox Balance and Inflammatory Signaling in Melanocytes and Melanoma Cells Irradiated with UVA

#### 4.2.1. Melanocytes

Both the literature and the present findings suggest that UVA radiation, by modulating signaling pathways related to oxidative stress, inflammation, and immune evasion, may contribute to the neoplastic transformation of melanocytes and melanoma progression [3]. Consequently, considerable attention has been directed toward identifying bioactive compounds capable of protecting against UVA-induced damage and restoring cellular homeostasis in melanocytes without promoting melanoma cell survival. Naturally occurring compounds are of particular interest due to their generally lower toxicity compared to synthetic agents [67]. In recent years, research on phytocannabinoids derived from *Cannabis sativa* L., especially CBD and CBG, has intensified [11].

The present study demonstrates that CBG exerts a cytoprotective effect in melanocytes, particularly under non-irradiated conditions, by reducing oxidative stress and enhancing pNrf2 expression. This was associated with increased levels of Nrf2-dependent antioxidants, including HO-1, SOD1, SOD2, and components of the GSH- and Trx-dependent systems. Consequently, reduced ROS levels and decreased lipid peroxidation products (including 8-iso-PGF2α and 4-HNE) were observed, leading to diminished modification of metabolically important proteins. Both CBG and CBD, administered individually or in combination, also attenuated UVA-induced activation of prooxidant enzymes. Among the analyzed compounds, CBG showed the strongest effect, suggesting its potential role in restoring redox homeostasis in irradiated melanocytes. Consistent with previous reports indicating that TrxR supports melanocyte homeostasis and DNA integrity under oxidative stress [68], the observed upregulation of HO-1 further suggests that CBG may promote cytoprotective heme metabolism [69].

UVA exposure not only induces oxidative stress but also promotes a proinflammatory environment. Post-treatment with CBG or the CBG + CBD combination normalized NF-κB levels and its downstream target TNF-α, which are associated with pro-oncogenic signaling pathways [70]. This shift toward a more antioxidant state was accompanied by reduced lipid peroxidation and protein modification, supporting the regenerative potential of phytocannabinoids in oxidatively damaged cells. It is clear that phytocannabinoids affected levels of signaling proteins/transcription factors such as Nrf2 and NF-κB; however, their real effect on gene expression is still not fully explored. Further studies are warranted to clarify the biological significance and therapeutic potential of these findings.

Overall, phytocannabinoids particularly CBG stabilized melanocyte metabolism under both basal and UVA-exposed conditions. These findings are consistent with previous studies showing that CBG reduces UV-induced oxidative and inflammatory responses in skin cells and protects membrane phospholipids more effectively than CBD [71,72,73]. Additionally, CBG has been reported to suppress inflammatory signaling and cytokine production more efficiently than CBD in various cell types [74,75]. The distinct biological effects of these phytocannabinoids may result from structural differences influencing their reactivity and interaction with cellular targets [76].

#### 4.2.2. Melanoma

Literature data indicate that alterations in metabolic pathways regulating redox balance in melanocytes toward a pro-oxidant and pro-inflammatory state promote their transformation into melanoma and drive metabolic reprogramming in melanoma cells [77]. These observations are consistent with the present findings on the response of melanocytes and SK-MEL-5 cells to UVA radiation. Cannabidiol (CBD) has been shown to reduce the viability of melanoma cell lines via activation of G protein-coupled receptors, including CB1 and CB2, as well as by modulating the expression of other receptors such as TRPV1 and PPARα, thereby influencing redox and inflammatory responses [15]. However, the present results provide a more nuanced view of metabolic interactions in melanoma cells under combined exposure to UVA radiation and phytocannabinoids.

Melanoma cells exhibit a metabolic shift toward a more oxidative phenotype [61], which is supported by the significantly elevated NOX activity and ROS levels observed in this study compared to melanocytes. Under basal conditions, phytocannabinoids reduced ROS levels in melanoma cells, where oxidative stress is driven by mechanisms such as PI3K/AKT hyperactivation [78], hypoxia [79], metabolic reprogramming [80], and increased activity of pro-oxidant enzymes (e.g., NOX, NOS) [81,82]. Moreover, CBG and the CBG + CBD combination attenuated UVA-induced ROS generation, suggesting a potential modulation of pro-oxidant signaling pathways rather than a direct indication of reduced malignancy. This redox modulation may contribute to decreased proliferation and invasiveness of melanoma cells. Previous studies have shown that CBD reduces melanoma cell viability by disrupting mitochondrial integrity and inducing apoptosis [14,15]. In parallel, CBG has been reported to modulate immune-related pathways, including inhibition of CSF-1 secretion, suppression of MDSC expansion, and enhancement of anti-PD-L1 therapy, ultimately promoting apoptosis in glioblastoma models [16,83], which may partially translate to melanoma biology.

In this study, CBD and the CBD + CBG combination reduced the expression of pNrf2 and HO-1, as well as the activity of thioredoxin-dependent systems in SK-MEL-5 cells, which may further limit melanoma cell survival. The differential effects of CBD and CBG on SOD1 activity following UVA exposure may be attributed to their distinct molecular structures and conformational properties, which influence their interactions with protein targets. Additionally, UVA radiation itself may alter the structural organization of SOD1 complexes, particularly those involving copper and zinc ions, thereby affecting enzyme activity [84]. Nrf2 is known to interact with MAPK and MITF signaling pathways and can modulate EGFR expression, influencing melanoma proliferation and stress responses [85,86]. Therefore, phytocannabinoid-induced modulation of antioxidant pathways may differentially affect ROS-mediated signaling in melanoma cells.

The observed increase in glutathione levels and glutathione reductase activity, particularly following CBD and CBD + CBG treatment, indicates activation of antioxidant defense systems. This may contribute to the protection of cellular components, including membrane lipids. Phytocannabinoids also influence the thioredoxin system, and TrxR overexpression has been suggested to modulate metabolic pathways, including glycolysis, under oxidative stress conditions [68]. These findings support the notion that oxidative stress creates a metabolic environment conducive to cancer progression, while phytocannabinoids exert complex, potentially pleiotropic effects on melanoma cell metabolism. Oxidative stress, especially following UVA exposure, promotes the interaction of ROS with polyunsaturated fatty acids, leading to lipid peroxidation [87,88]. Elevated levels of 4-HNE in dysplastic nevi compared to benign lesions have been linked to melanomagenesis through DNA adduct formation [89,90]. In this study, phytocannabinoids particularly CBG reduced lipid peroxidation and the formation of 4-HNE–protein adducts, indicating partial mitigation of oxidative damage. Since lipid peroxidation products may also contribute to immune evasion mechanisms in melanoma [91], their reduction may have additional biological relevance.

Nrf2 is known to interact antagonistically with NF-κB, a central regulator of inflammation and tumor progression [92]. While phytocannabinoids increased NF-κB expression in non-irradiated melanoma cells, treatment with CBD or CBG following UVA exposure reduced NF-κB levels compared to UVA alone. NF-κB activation in melanoma is commonly associated with BRAF mutations, PI3K/AKT pathway dysregulation, and PTEN loss, contributing to tumor survival and resistance through anti-apoptotic signaling [70,93,94,95]. The observed modulation of the Nrf2–NF-κB axis may therefore reflect a shift toward reduced inflammatory and pro-tumorigenic signaling. Increased glutathione levels following CBD and CBG treatment may also indicate activation of the pentose phosphate pathway, providing NADPH required for antioxidant defense and further contributing to suppression of NF-κB activity.

Despite the potential translational relevance of these findings, the results should be considered preliminary. The study was conducted using a single dose of UVA radiation and single concentrations of phytocannabinoids, selected experimentally, which do not capture the full spectrum of possible metabolic responses in the analyzed cells. Consequently, dose–response relationships could not be assessed. In addition, the study was limited to in vitro experiments using cultured melanocytes and a single melanoma cell line. Although this approach enables controlled analysis of redox and inflammatory signaling, it does not reflect the complexity of skin tissue, including intercellular interactions and the influence of systemic factors. Therefore, further validation in animal models and clinical settings is required. It should also be noted that chronic UVA exposure and prolonged phytocannabinoid treatment may induce responses that differ substantially from those observed under the experimental conditions, particularly in metabolically adaptable melanoma cells. Moreover, while phytocannabinoids modulated ROS levels and antioxidant systems, their effects on key functional outcomes such as cell proliferation and apoptosis were not assessed. Further studies are therefore necessary to determine the biological significance and therapeutic potential of these observations.

## 5. Conclusions

The primary risk factor for melanoma development is excessive cutaneous exposure to ultraviolet radiation, which induces metabolic alterations in melanocytes and contributes to carcinogenesis. Accordingly, the identification of novel strategies for melanocyte protection and melanoma prevention and treatment remains a key objective in dermatological and oncological research. The observed stabilizing effect of phytocannabinoids on the redox homeostasis of UVA-irradiated melanocytes is particularly relevant, as it may reduce conditions that favor neoplastic transformation. However, the partial protective and potentially regenerative effects of phytocannabinoids observed in SK-MEL-5 cells suggest that their topical application in cases of suspected malignant lesions should be approached with caution. The present findings also highlight the need for further studies, particularly at the proteomic and lipidomic levels, to better understand the molecular mechanisms underlying these effects.

## Data Availability

The original contributions presented in this study are included in the article and Supplementary Materials. Further inquiries can be directed to the corresponding authors.

## Supplementary Materials

The following supporting information can be downloaded at: https://www.mdpi.com/article/10.3390/antiox15060690/s1, Table S1: Comparison of the effects of phytocannabinoids (CBG, CBD, and CBG + CBD) on redox balance parameters and markers of oxidative stress and inflammation in control and UVA-exposed melanocytes and melanoma cells; File S2: Supplementary Figures; Figure S1: The morphology of the melanocytes and SK-MEL-5 cells; Figure S2: The viability of the melanocytes and SK-MEL-5 cells. Results obtained using MTT assay; Figure S3: The effect of phytocannabinoids on cells proliferation measured as protein biosynthesis intensity in the melanocytes and SK-MEL-5 cells; Figure S4: Effects of phytocannabinoids on cell death in melanocytes and SK-MEL-5 cells.

## Abbreviations

The following abbreviations are used in this manuscript:

4-HNE	4-hydroxynonenal
8-isoPGF2α	8-iso prostaglandin F 2α
BRAF	proto-oncogene B-Raf
CB1	cannabinoid receptor 1
CBD	cannabidiol
CBG	cannabigerol
CO	carbon monoxide
CSF-1	colony-stimulating factor 1
EGF	epidermal growth factor
EGFR	epidermal growth factor receptor
FAK	focal adhesion kinase
GPx	glutathione peroxidase
GSH	glutathione
GR	glutathione reductase
GST	glutathione S-transferase
HO-1	heme oxygenase 1
Keap1	Kelch-like ECH-associated protein 1
MAPK	mitogen-activated protein kinases
MDA	malondialdehyde
MITF	microphthalmia-associated transcription factor
MO-MDSC	monocytic myeloid-derived suppressor cells
mTOR	mammalian target of rapamycin kinase
NF-κB	nuclear factor kappa-light-chain-enhancer of activated *B* cells
NOS	nitric oxide synthases
NOX	nicotinamide adenine dinucleotide phosphate oxidase
Nrf2	nuclear factor erythroid 2-related factor 2
PI3K	phosphoinositide 3-kinase
PKB	protein kinase B
PPARα	peroxisome proliferator-activated receptor alpha
PTEN	phosphatase and tensin homolog
PUFAs	free polyunsaturated fatty acids
ROS	reactive oxygen species
Sesn2	sestrin 2
sFRP2	secreted protein 2
SOD	superoxide dismutases
TAM	tumor-associated macrophage
TNF-α	tumor necrosis factor alpha
TRPV1	the transient receptor potential cation channel subfamily V member 1
Trx	thioredoxin
TrxR	thioredoxin reductase
UV	ultraviolet
VEGF	vascular endothelial growth factor
XO	xanthine oxidase
