# Supplementary material for: Metabolic Responses of Melanocytes and Melanoma Cells to UVA Radiation and Phytocannabinoids Exposure
Source: Antioxidants (Basel). 2026 May 30;15(6):690. doi: 10.3390/antiox15060690 (PMC13295252; doi:10.3390/antiox15060690)
Supplement: Supplementary file 1 [file antioxidants-15-00690-s001.zip › Supplementary Materials-file S1.pdf]

Table S1. Comparison of the effects of phytocannabinoids (CBG, CBD, and CBG+CBD) on redox balance parameters and markers of oxidative stress and inflammation in control and UVA-exposed melanocytes and melanoma cells.

	**MELANOCYTES**							**SK-MEL-5 CELLS**						
	No radiation			UVA				No radiation			UVA			
	CBG	CBD	CBG+CBD	UVA	CBG	CBD	CBG+CBD	CBG	CBD	CBG+CBD	UVA	CBG	CBD	CBG+CBD
	vs control			vs control	vs UVA			vs control			vs control	vs UVA		
**NOX**	—	—	↓	↑	↓	↓	↓	—	↓	↓	↑	↓	↓	↓
**XO**	—	—	—	↑	—	—	↓	—	↓	↓	↑	↓	—	↓
**ROS**	↓	—	↓	↑	↓	↓	—	↓	↓	↓	↑	↓	—	↓
**pNrf2**	↑	—	—	↑	↑	—	—	—	—	—	↑	—	↓	↓
**HO-1**	↑	—	—	↑	↑	↓	—	—	—	—	↑	↓	↓	↓
**SOD-1**	↓	—	↓	↓	↑	↑	↑	—	—	↑	↓	—	↓	↑
**SOD-2**	↓	—	↑	↓	↑	↑	↑	↓	↑	↑	↓	↑	↑	↑
**GSH**	—	—	—	↓	—	—	—	—	↑	—	↓	↑	↑	↑
**GPx**	↑	—	↑	↓	↑	↑	↑	↓	↓	↓	↑	—	—	—
**GR**	—	—	↑	↓	↑	↑	↑	↑	—	↑	↑	—	↑	↑
**Trx**	↑	—	↑	↑	↑	—	—	—	—	—	↑	—	↓	↓
**TrxR**	—	↑	↑	↑	↑	—	↑	↑	—	↑	↑	↓	↓	↓
**4-HNE**	—	—	↓	↑	—	—	—	↓	↓	↓	↑	↓	↓	↓
**4-HNE-protein**	—	—	—	↑	↓	↓	↓	—	↓	↓	↑	↓	↓	↓
**8-isoPs**	—	—	—	↑	↓	↓	↓	—	—	—	↑	↓	↓	↓
**NF-κB (p52)**	—	—	↑	↑	↓	↓	↓	—	—	↑	↑	—	↓	↓
**NF-κB (p65)**	—	—	—	↑	↓	↓	↓	—	—	↑	↑	—	↓	↓
**TNF-α**	↑	—	—	↑	—	—	—	↑	—	—	↑	—	—	—
