# Supplementary material for: Metabolic Responses of Melanocytes and Melanoma Cells to UVA Radiation and Phytocannabinoids Exposure
Source: Antioxidants (Basel). 2026 May 30;15(6):690. doi: 10.3390/antiox15060690 (PMC13295252; doi:10.3390/antiox15060690)
Supplement: Supplementary file 1 [file antioxidants-15-00690-s001.zip › Supplementary Materials-file S2.pdf]

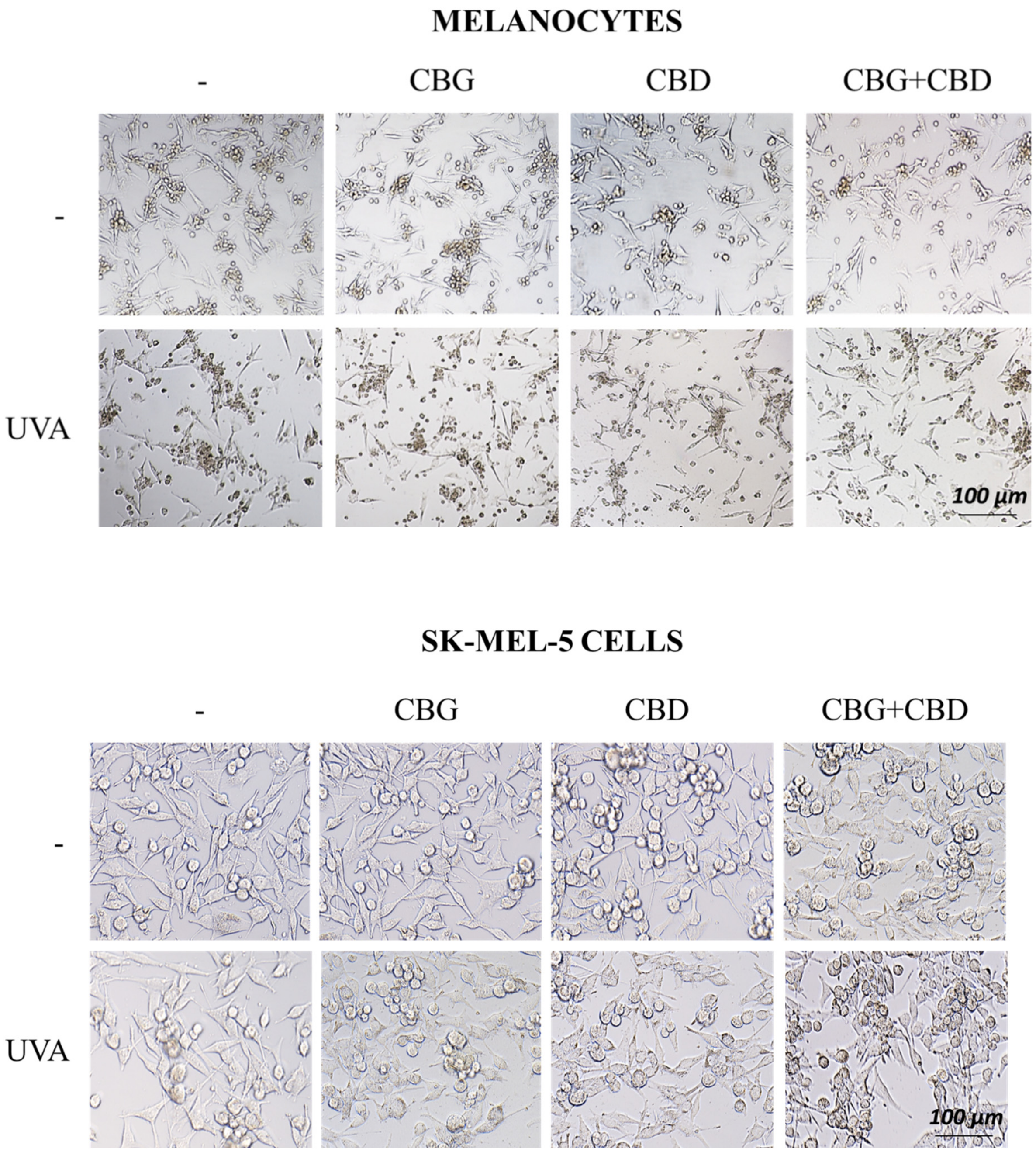

Figure S1. The morphology of the melanocytes and SK-MEL-5 cells. Photos obtained using light microscope NikonEclipse Ti combined with MagingSource camera operated by Nikon's NIS-Elements imaging software version 5.30.02 (Nikon Instruments Inc., Melville, NY, USA).

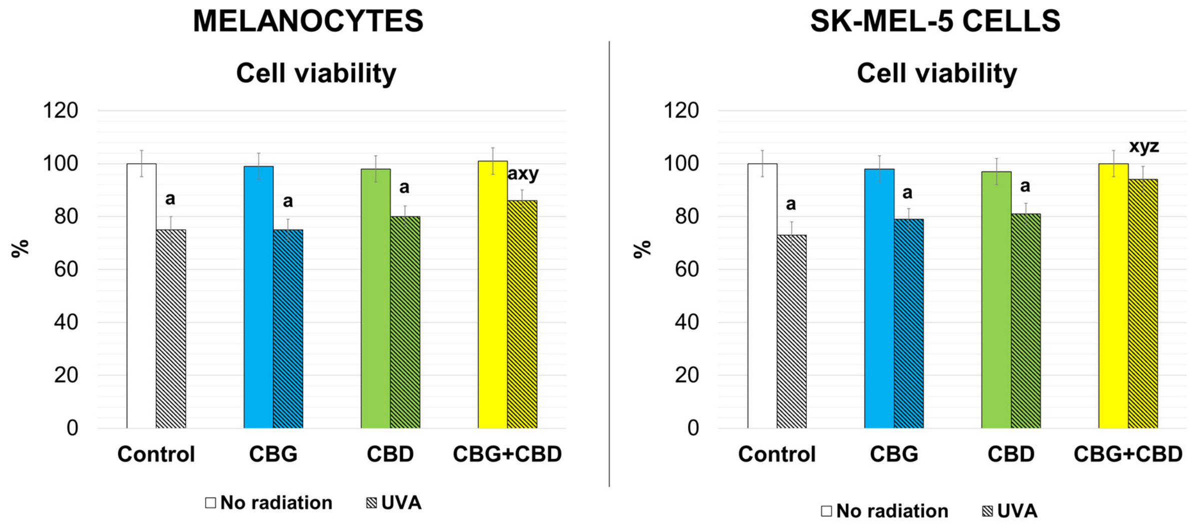

Figure S2. The viability of the melanocytes and SK-MEL-5 cells. Results obtained using MTT assay.

The mean±SD values (n=5) are presented with statistically significant differences: a–vs.control group; b–vs.CBG group; c–vs.CBD group; x–vs.UVA group; y–vs.UVA+CBG group; z–vs.UVA+CBD group; $p<0.05$.

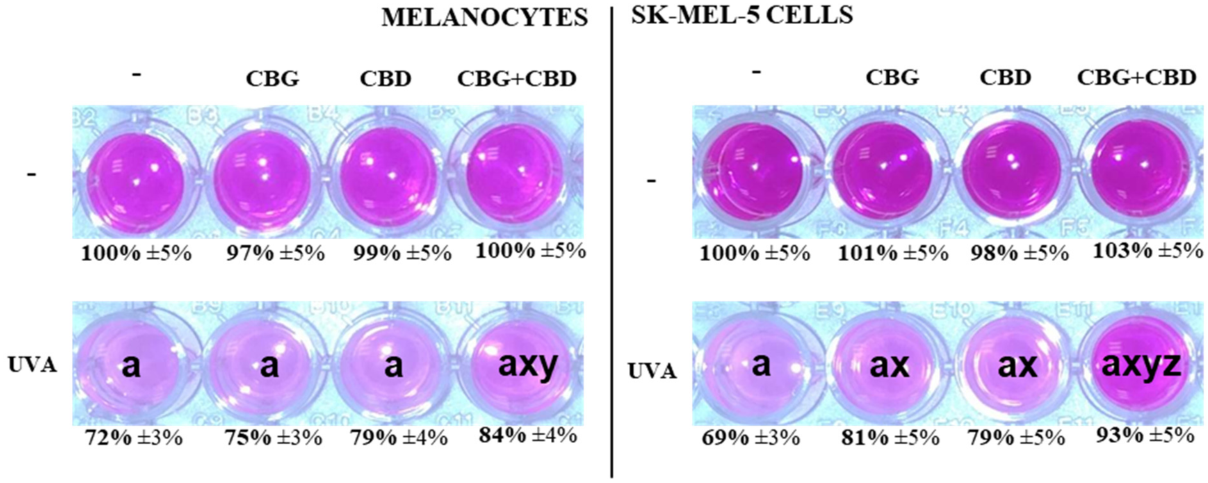

Figure S3. The effect of phytocannabinoids on cells proliferation measured as protein biosynthesis intensity in the melanocytes and SK-MEL-5 cells. Results obtained using SRB assay.

The mean±SD values (n=5) are presented with statistically significant differences: a–vs.control group; b–vs.CBG group; c–vs.CBD group; x–vs.UVA group; y–vs.UVA+CBG group; z–vs.UVA+CBD group; $p<0.05$.

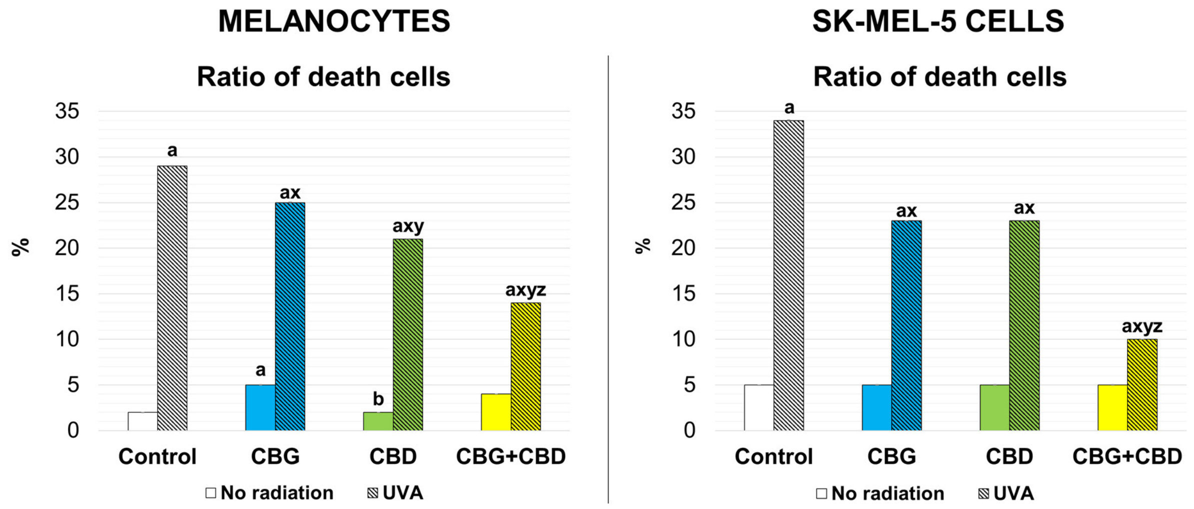

Figure S4. Effects of phytocannabinoids on cell death in melanocytes and SK-MEL-5 cells. Results obtained basing on dead cells staining with trypan blue.

The mean±SD values (n=5) are presented with statistically significant differences: a–vs.control group; b–vs.CBG group; c–vs.CBD group; x–vs.UVA group; y–vs.UVA+CBG group; z–vs.UVA+CBD group; $p<0.05$.
